# What Price Are We Willing to Pay for the Dream of Equal Justice?^†^

**DOI:** 10.1093/ojls/gqab002

**Published:** 2021-09-21

**Authors:** Andrew Higgins

**Keywords:** costs and funding, legal profession, equality, procedural justice

## Abstract

The injustices wrought by unequal access to the legal system pose a direct threat to the rule of law, yet such injustices are widespread in England and elsewhere. Lawyers regularly criticise governments for a lack of funding for the legal system, but the private market for delivering legal services receives much less scrutiny. A private market for legal resources is antithetical to equal justice because it makes the outcome of cases turn on arbitrary factors such as wealth. The solution, according to Wilmot-Smith in his book *Equal Justice*, is to socialise the allocation of legal services so that the rich cannot buy the best lawyers, and to prevent them from contracting out of this public system by making private arbitrations unenforceable. This review article argues that Wilmot-Smith’s thesis is persuasive, but there might also be second-best solutions that could deliver greater legal equality at lower cost.

## 1. Introduction

Virtually all legal scholars, numerous constitutions and most people acknowledge the importance of equal treatment according to law and equal access to the justice system. Yet anyone who has come into contact with the legal system in England knows the reality is anything but equal justice for all. Many people cannot access the system due to the unaffordability of legal representation, while many are forced to, or attempt to, navigate the system on their own, with limited or no legal assistance. In the civil sphere, there is the further, logically distinct problem that even if legal assistance is affordable, its cost is so disproportionate to the financial value of the rights in dispute that it would be economically irrational to retain a lawyer.

This would be problematic enough if it was any other public service that people had difficulty accessing, but the problems are deepened in a justice system where disputes about rights are usually decided via an adversarial process where there is another party actively advocating a position that is hostile to your legal interests. This other party is often the state—with all the resources and information-gathering powers available to it—or ‘lawyered up’ large corporations. The gross inequality of arms in these adversarial contests does not inevitably result in unjust outcomes, but there is a high probability that the risk of wrongful judgments is borne disproportionately by the poor, because the rich are able to buy better-quality lawyers who maximise the chances of obtaining favourable judgments, regardless of the merits of their claims.

In *Equal Justice: Fair Legal Systems in an Unfair World*, Wilmot-Smith sets out a powerful case for socialising access to the justice system and the legal services market more broadly, in line with a commitment to delivering equal justice. Section 2 of this review gives a brief overview of the book and initial scholarly and legal reaction to it, section 3 examines the logic and the tension in Wilmot-Smith’s argument for equal justice in imperfect legal systems, section 4 considers what priority should be given to the pursuit of equal justice and sections 5 and 6 consider whether second-best solutions might be able to significantly lower the risk of unequal justice but without the costs involved in Wilmot-Smith’s model.

## 2. Brief Overview of the Book

To begin, a note on methodology is warranted. Wilmot-Smith does not construct his argument from empirical experience at the coalface of the justice system, although the book is peppered with interesting historical and current examples throughout. Instead, he develops a philosophical account of the requirements of a just justice system combining legal and political philosophy. This is both an admirable strength and a potential weakness of the book. Wilmot-Smith’s choice of methodology is justified by the outcome—an unrelenting and powerful critique of norms that are so widespread in the legal community, particularly among practising lawyers, they are hardly ever challenged: (i) that legal justice can never be rationed, an idea that presupposes that law and lawyers are the sole exception to the distributive justice dilemma of how best to allocate benefits and burdens in a world of scarce resources; and (ii) relatedly, everyone should be free to engage lawyers on terms they choose in a private market.

Wilmot-Smith’s critique of a private legal services market, and its tendency to undermine the central purpose of a legal system, is compelling. Markets inevitably lead to ‘arbitrariness in the distribution of the benefits and burdens of legality’.^1^ While it is difficult to trace the effect of an individual lawyer on a particular case, unless the market for private legal services is wholly dysfunctional, it is plausible that those lawyers who can command the highest fees are more likely to improve a client’s prospects.^2^ A market in legal resources is therefore ‘a bad way of distributing legal resources. It fails even to approximate to the ideal of equal justice, making outcomes turn on antecedent wealth rather than the merits of the claim.’^3^ This, in turn, risks undermining the rule of law and exacerbating inequality in the real world, as the rich are able to enforce their rights when they choose, whilst avoiding their obligations without sanction.^4^ The purpose of a legal system is undermined if the domination it seeks to prevent is reconstituted by way of control of the law’s coercive institutions. Accordingly, ‘the justice benefits and burdens of legality should be equally shared, so far as that is possible’.^5^ This equal benefit and burden sharing is what Wilmot-Smith defines as equal justice.

Wilmot-Smith recognises that equal justice is, in reality, impossible, partly because the legal system is itself imperfect. To make any inequality justifiable to those who lose out, the inequality must issue from a fair procedure. While Wilmot-Smith does not engage in a traditional analysis of what constitutes a fair procedure—the various rules and obligations that make up litigants’ due process rights, a point to which I will return—he does examine in-depth the level and allocation of resources needed to make those processes fair. He begins on the conventional ground that all persons should have access to a basic level of legal resources, but then radically upgrades the argument, stating that in the context of legal justice, ‘enough’ resources means ‘roughly equal’ resources.^6^ Moreover, this entitlement to ‘roughly equal’ resources applies not just to parties in individual cases, but also equal access to legal resources across the legal system. Wilmot-Smith would only permit inequality in distribution of legal resources if it would not leave the poor worse off relative to an alternative arrangement. To give effect to this commitment to equal justice, Wilmot-Smith would not only socialise the delivery of legal representation to allocate evenly qualified lawyers to the resolution of disputes^7^, but also prevent the rich from contracting out of this socialised system by resorting to private arbitration and ‘siphoning’ off all the best lawyers and judges in the process.

Wilmot-Smith provides a powerful critique of the doubly inegalitarian nature of arbitration. Wealthier persons can get better arbitrators, and access to dispute resolution, that are not available to others.^8^ To these flaws, we can add a third: viewed as a manifestation of the contractual intention of the parties, but where wealthier parties can dictate the terms on which anyone contracts with them, they can use arbitration clauses to force the counterparty to effectively surrender the option of enforcing their legal rights. This is achieved through the incorporation of arbitration clauses that make it unlawful to litigate in court, but also expensive and/or economically irrational to arbitrate the dispute.^9^ For the millions of consumers and small businesses who have no practical choice but to sign contracts with compulsory arbitration clauses, making arbitration truly voluntarily would be a welcome development. Forcing these disputes back into the courts would come with the added benefits of public legal adjudication, including transparency and clearer precedent. Wilmot-Smith makes an additional empirical claim—that allowing the rich to contract out of public services has a corrosive effect on the quality of the public option—to support his proposal that the state ‘should refuse to lend its hand to the enforcement of private dispute resolution mechanisms’.^10^

Recognising the radical nature of his proposals, Wilmot-Smith dedicates much of the rest of his book to defending them against potential objections, and explaining how a socialised legal service might work in practice. He dismisses freedom-of-contract objections, concerns about state paternalism and the potential undermining of lawyers’ independence. While Wilmot-Smith claims that there are no autonomy-based objections to his model,^11^ he does accept that some of these concerns have implications for how his model should be implemented. He argues that clients should still be free to choose their own lawyer, just not the terms of payment. In addition, the risk that centralisation of the process for allocating legal resources would concentrate too much power in government can also be managed through proper adherence to the separation of powers so that the allocation could be decided by independent lawyers administering guidelines developed by governments.^12^ In relation to the funding of the system, Wilmot-Smith, unsurprisingly, advocates a system of general taxation, however he also recognises that modest court fees can play a useful role in weeding out frivolous or vexatious disputes.^13^

The book has already received high praise from distinguished commentators, as well as some criticism. If all reviews, no matter how positive or negative, take the format ‘this is an excellent book, but …’, I should make clear that while much of this review is dedicated to the ‘but’ section, my assessment of the book is that the excellent adjective should be underlined, and my buts are very much of the small b variety. Like Lord Neuberger in his comments on the book, I anticipate that most of the questions for Wilmot-Smith will relate not to his critique of a private legal service market, but his proposed solutions to the injustices it causes.

A book that calls for a radical overhaul of the legal system would, if realised, create new winners and losers. We need to bear that in mind when considering responses from the legal community who are likely to have benefited, to varying degrees, from the current legal system. Some criticisms of Wilmot-Smith’s book are remarkably transparent in this regard—he is even criticised for failing to consider the negative impact on law alumni donations to university endowments.^14^ These ‘civilisation will end’ criticisms have echoes of the extreme arguments advanced against public healthcare in the United States, or the creation of the National Health Service (NHS) in the UK. Yet arguably the greatest case for Wilmot-Smith’s proposals can be found in the overwhelming public support for the NHS. Indeed, public support for the NHS is so strong that even right-wing politicians vow to increase funding for it on their campaign buses when encouraging people to vote for radical constitutional change.^15^ The public seem to like socialisation of vital public services because they are built on the principle that everyone is entitled to equal treatment regardless of circumstances. While all acknowledge, including Wilmot-Smith, that there is not similar public support for a National Legal Service, the greatest achievement of Wilmot-Smith’s book is to outline a sophisticated philosophical case for an exclusively public legal system, one designed to distribute the benefits and burdens of legality equally.

Personally, I would sign up to Wilmot-Smith’s model for equal justice with just a few outlier qualifications. Nonetheless, some of the philosophical and practical criticisms of the book have merit, and in examining key pillars of Wilmot-Smith’s case for equal justice, I will also outline a case for second-best solutions^16^ that could go a substantial way to delivering the equality Wilmot-Smith argues for, but without the cost involved in his proposed reforms.

## 3. Equal Justice in Imperfect Legal Systems

Wilmot-Smith dedicates a significant amount of copy to philosophical debates on equality, including objections to certain versions of it. For readers interested in political philosophy, I would recommend an insightful review of *Equal Justice* by political philosopher, James Wilson.^17^ Two points from Wilmot-Smith’s discussion of equality stand out as having particular salience for the debates on how to fund and administer the justice system. The first is that equal justice is an ideal, and impossible in reality. All legal systems must strive for equal justice but none can never knowingly achieve it. The legal process is the quintessential example of a system of imperfect procedural justice, where there are independent criteria for a just outcome—including, most obviously, the correct application of the law to the true facts—but no means of guaranteeing that outcome.^18^ Accordingly, the study of legal procedures is an examination of which processes and evidence are best calculated to advance truth finding consistent with the other ends of the law.^19^ The capacity of these procedural and evidentiary rules to deliver just outcomes is not a focus of Wilmot-Smith’s book, but Wilmott Smith is fully conscious of the dilemmas and choices that the imperfect nature of legal systems throw up.

Setting up a legal system is morally risky.^20^ Law restricts people’s ability to take just actions in favour of submitting to formal processes that carry an unavoidable risk of error. Hence there is a need to evenly allocate the benefits and burdens of legality, including the chance that a court will deliver incorrect judgments. All agree that these chances should not depend on arbitrary factors. Unfortunately, we can never be sure whether arbitrary factors have influenced judgments because we have no meta-test to assess the correctness of individual decisions. In some instances, aggregate statistics are so stark in the differential treatment they reveal, in the context of race for example, that it is incontrovertible that the benefits and burdens of legality are not being distributed equally.^21^ But aggregate statistics cannot tell us anything about the justice of individual cases, and for this reason the courts are reluctant to use statistics to determine the outcome of individual cases for fear that it might cause an injustice in the instant case.

There are also more practical, mundane reasons why achieving equal justice is virtually impossible. As Wilmot-Smith recognises, numerous factors can influence the outcome of cases, including, as the sports commentators like to say, ‘who wants it more’. Wilmot-Smith’s proposals are dedicated to preventing parties from buying the better legal team, but that cannot prevent parties from investing more time in preparing their case, with the possibility that this might lead to a wrong judgment in their favour. Yet trying to limit party effort would be draconian, and difficult to achieve, as Wilmot-Smith acknowledges.

The basic point is that we cannot guarantee equal justice in practice. One solution to the inability to measure equal justice (in outcomes) is to adopt the fairest procedures possible for delivering justice, including making any adversarial process between parties as equal as possible. This is because deviations from equal justice can only be justified if they issue from a fair procedure.^22^ But an alternative, less ambitious approach is to embrace a proportionality analysis which strives for procedures that are as fair as possible, taking into account the burdens of a higher risk of error and the costs of seeking to minimise that error. Given that the ideal of equal justice is unobtainable, all societies must weigh up distributive justice considerations when designing a justice system. How much are we prepared to invest to achieve equal justice knowing we cannot guarantee it in practice, will never know whether we have achieved it, and there are lots of worthy things we could do with the resources that we could otherwise dedicate to its pursuit?

Interestingly, Wilmot-Smith begins his analysis of the implications of the impossibility of delivering equal justice by arguing for the second approach—the creation of a ‘fairness floor’ which guarantees a basic level of resources.^23^ However, when ‘excavating’ that floor he updates his argument to an entitlement to roughly equal resources in line with a commitment to equal justice.^24^ A crucial caveat, however, is that Wilmot-Smith’s analysis is too sophisticated to amount to a rallying call for an unlimited commitment to achieving justice for all. He recognises that even legal justice as administered by the courts has a distributive justice dimension built into it: no one is entitled to an unlimited amount of resources to vindicate their legal rights. This point is neatly made by Dworkin in ‘Principle, Policy, Procedure’.^25^ Equal justice is not the same thing as equal resources.

Nonetheless, Wilmot-Smith is prepared to go a long way to ensure that the possibility of equal justice is not undermined by the reality of unequal resources. Some key reasons he offers for the gold-plated fairness of a mandatory, public system is that if no one can buy their own judges—even if it be efficient—then the logic should also apply to lawyers who are equally engaged in the process of delivering justice. More fundamentally, Wilmot-Smith draws attention to both the risk of unjust outcomes in individual cases and the risk of systemic inequality, where some litigants are allocated a basic level of legal resources while others can get the best legal teams money can buy.^26^ The spectre of hopelessly mismatched legal teams (be it in talent, time or resources) skewing the outcome of cases creates an obvious rule of law problem; it increases the risk of might trumping right. But rule-of-law concerns also arise where legal systems do not treat like cases alike. Crucially, unlike the David v Goliath suit where we suspect, but cannot verify, that might has trumped right, in the case of inconsistent outcomes *across* like cases, the injustice is undeniable. While we cannot know which of the inconsistent cases is wrong, we know for sure one of them must be.

Both Wilmot-Smith and I agree that inconsistency cannot stand in a just justice system, but we have developed different—though not incompatible—solutions to the problem. I have argued that a legal system must develop procedures to avoid the risk of inconsistency, focusing, in the civil context, on mandatory aggregation procedures such as joinder and collective redress rules.^27^ If cases giving rise to common questions of fact and law are dealt with in the one proceeding, the risk of inconsistency falls away. The idea of mandatory legal processes has radical implications for the organisation of the justice system, given the frequency with which courts deal with cases raising common issues. Wilmot-Smith’s radical solution, by contrast, focuses not on the legal procedures that could give rise to inconsistency, but rather on the legal system’s players. Put crudely, if we can even out the resources that can be dedicated to cases, we are likely to even out the outcomes that litigants or criminal defendants obtain from those cases. This is not a complete solution because it does not tackle the risk of inconsistent outcomes directly. Two sets of equally talented lawyers and judges involved in different proceedings deciding the same question can still, acting in good faith, arrive at different outcomes. The unequal treatment problem persists: the justice system has delivered outcomes one of which is *known* to be wrong, even if the risk of injustice, which did in fact materialise, was borne equally by the parties in Wilmot-Smith’s model.^28^ But Wilmot-Smith’s model does have the attraction of being potentially more workable than my own, certainly in the criminal sphere, where joinder is not practical and could be unjust in the context of jury trials.^29^

More radical still, Wilmot-Smith’s mission for equality is not confined to evening up the scales in individual cases or treating like cases alike. The scope of Wilmot-Smith’s ambition becomes clear when he commits to equality of arms *and* Rawlsian institutional fairness so that the justice system produces the maximum amount of equality possible when compared with an alternative arrangement.^30^ Maximising the position of the worst off is not the same as equal distribution. Similarly, equality of arms does not require the same level of legal talent to be available for all legal disputes.^31^ But when these two equality objectives are combined, this leaves Wilmot-Smith with little room for manoeuvre when designing a justice system. Wilmot-Smith would only allow the rich to buy better lawyers, even in disputes between themselves, if that would not leave the poor worse off. And according to him, it generally would leave them worse off because it results in the rich siphoning off the best legal minds, and potentially buying up the time of the best judges.

None of this is meant as criticism of Wilmot-Smith’s analysis; there is a clear logic to it, but there is also an obvious tension in dedicating so much effort to achieving roughly equal legal resources when the ultimate aim—equal justice—is an unobtainable ideal. When deciding whether we should make that effort, it is worth considering the priority we ought to give to the pursuit of equal justice and whether there are other, marginally inferior, methods (second-best solutions) for pursuing equal justice that would involve substantially lower costs. It is to these questions that I now turn.

## 4. Distributing Justice—What Priority Should We Give to Equal Justice?

In his book, Wilmot-Smith makes clear that the case for equal justice is theoretically distinct, and severable from, the case for equality more generally. This leads us to consider the priority that should be given to justice over other interests that states could legitimately pursue. Here Wilmot-Smith’s thesis is conventional from a lawyer’s perspective, but where I part company from him slightly.

Wilmot-Smith begins by rightly acknowledging that legal resources are scarce and that ‘there is not enough court time for all to have their day in court; there are not enough lawyers for everyone to have one for every legal problem’.^32^ However, for him, legal systems should have a priority claim on society’s resources. This is because legal systems are not just another consumer good or even one amongst many vital public services that states should support. Justice has ‘a special value’ which is ‘carried through to legal institutions’.^33^ In this claim, he has very distinguished legal company. In the *Unison* case, the UK Supreme Court struck down unaffordable and often disproportionate Employment Tribunal Fees. In delivering the Court's judgment, Lord Reed declared that legal systems are no ordinary public service:

In order for the courts to perform [their] role, people must in principle have unimpeded access to them. Without such access, laws are liable to become a dead letter, the work done by Parliament may be rendered nugatory, and the democratic election of Members of Parliament may become a meaningless charade. That is why the courts do not merely provide a public service like any other.^34^

For most political philosophers—from libertarians to those with more social democratic outlooks—a just legal system is a cornerstone of a just political order. Perhaps counter-intuitively, the need for the state to support a just legal system arguably increases in importance the more one believes in a minimalist state. Given libertarians’ defence of justice in individual transactions^35^, generally irrespective of background conditions, and their view that the state’s primary role should be to protect the integrity of those transactions, such as protection of property and contracts, they could, perhaps should, support the legal system having a priority claim on substantial public funds.^36^ The integrity of the libertarian position is undermined if the rich can bargain their way to greater benefits *and* purchase their way out of the unwanted consequences of their own agreements by buying better lawyers, or renege on agreements safe in the knowledge that the other party has no means of redress. Wilmot-Smith powerfully critiques the idea that a market in private legal services could be defended as just even if it produces inequalities, provided the individual transactions are themselves defensible. Whether people are in a position to afford legal insurance, or can pay for legal representation at cost, ‘is irrelevant as a matter of justice to the litigants’ dispute. One individual’s decision not to purchase legal insurance does not make them more deserving of suffering an injustice, or less deserving of the benefits of legality.’^37^ The aim of a justice system is to eliminate these sorts of arbitrary factors affecting the outcome of cases.

Wilmot-Smith nails his ideological colours firmly to a Rawlsian contractualist mast, arguing that the inequality that results from voluntary market transactions can only be justified where they occur in conditions of ‘background justice’.^38^ An equal justice system is one of those background conditions: ‘there is no point prohibiting fraud if the defrauded cannot prove that advantage has been taken of them’.^39^ The larger point is that anyone who supports an ideal distribution of resources which tolerates inequality—whether it be in background conditions and/or inequality of outcomes through voluntary transactions—must subscribe to a system of equal justice to maintain the integrity of that distribution.

But the harder distributive question is how much should we invest in the pursuit of equal justice? It is not axiomatic that the pursuit of this unobtainable objective should take priority over other interests that states legitimately pursue. In the middle of a pandemic, even lawyers would be hard pressed to say that increasing legal aid should be governments’ top priority. Because justice and welfare considerations are severable, there are instances where not only might governments wish to prioritise welfare considerations over justice considerations (as they do all the time when fixing health, education and justice budgets), but promoting justice might also be detrimental to some welfare goals.

Public health is a good and topical example. Viruses that do not respect normative rules tend to highlight the normative choices made by societies—whose interests matter most. The challenge for lawyers who believe that law and legal rights have a special priority, and that this requires a corresponding right to unimpeded access to courts,^40^ is that these access rights sometimes have serious deleterious effects on community welfare. Relatively early in the pandemic, Lord Neuberger observed that the legal system could compound the economic slump caused by the pandemic and COVID-related restrictions. The risk is that in a time when a whole multitude of contractual obligations can no longer be met, strict enforcement of legal rights—from breach of contract to repossession claims—could trigger a downward economic spiral in which not just the defendant, but large swathes of the community go under. Lord Neuberger argued it was desirable, therefore, to provide companies with some ‘breathing space’.^41^ To achieve this, he called for a greater focus on alternative dispute resolution (ADR), which most commentators would agree is a sensible idea.^42^ But given the consensual nature of ADR, unless parties agree to compromise their rights, law makers will have to force people into a more enlightened position—whether by delaying their ability to enforce legal rights, compromising rights through statute or forcing parties into compulsory arbitration for that purpose.

In England, the Master of the Rolls introduced a moratorium on enforcement in housing possession cases, which was then subject to legal challenge on the grounds *inter alia* that it breached landlords’ right to a fair trial under article 6 of the European Convention of Human Rights.^43^ Article 6 includes an express right to trial within a reasonable time and an implied right of access to court.^44^ There are express qualifications on the right to a fair trial in article 6 relating to public hearings, and the implied rights that have been read into article 6 by the European Court of Human Rights (ECtHR) are also subject to necessary and proportionate exceptions. However, the express right to a fair trial within a reasonable time is not qualified. Where modifications to fair trial rights have been tolerated, typically on the grounds of national security, the ECtHR has stressed that those changes cannot impair the very essence of the right.^45^ The landlords’ position that a moratorium on repossession claims was breaching their article 6 rights was *prima facie* a strong one, even though the negative welfare consequences of allowing landlords to kick out (many thousands of) people from their homes, for circumstances that are wholly beyond the tenants’ control, are obvious. The landlords’ claim was only rejected on the grounds that the delay was short enough that it did not impair the essence of their article 6 rights. Subsequently, the government extended the delay on possession proceedings, including lengthening the notice period for such claims. Whatever the legal merits of the housing possession stays,^46^ the broader point is that sometimes legal justice can undermine community welfare. Thankfully, in more ordinary times, there is no direct conflict; it is not law versus welfare, but the challenge of choosing the right balance between different interests in a world of scarce resources. And law is not the only urgent concern.^47^

For all the reasons Wilmot-Smith powerfully articulates, no justice system worthy of the name could endorse the pursuit of unequal justice. However, we must recall that what is at stake when choosing between Wilmot-Smith’s model and second-best solutions is not a choice between equal justice or not, but what degree of *risk* of unequal justice in particular cases or contexts a society is willing to tolerate. Even Wilmot-Smith acknowledges that his case for equal justice is based on an entitlement to ‘roughly equal’ resources.^48^ The case for second-best solutions is that it might be possible to significantly lower the risk of unequal justice without the disruption and cost involved in the complete elimination of the private legal services market, including the arbitration market. It might also be possible to use some of the wealth generated by a private legal service market to invest in better legal services and/or other public services. Such second-best solutions cannot guarantee equal justice for all, but not even Wilmot-Smith’s ideal solution can do that, and they may be able to better achieve the Rawlsian goal of making the poor better off compared to alternative arrangements. In the next two sections, I will briefly set out how the gap between the risk of unequal justice that Wilmot-Smith’s model could deliver and that which exists with second-best solutions, may be narrower than it first appears.

## 5. Second Best Methods of Achieving Roughly Equal Justice

There are a range of procedural rules and funding innovations designed to promote, directly or indirectly, greater access to justice and greater equality between opposing litigants. Here I list some key procedures, but it is not an exhaustive list. There are commercial funding agreements, often referred to as contingency fees or damages-based agreements, that give prospective litigants access to the capital and lawyers needed to successfully prosecute their actions, often against very well resourced corporate defendants.^49^ There are fixed cost rules, very common in civil law jurisdictions, that provide litigants with concrete certainty about their adverse costs liability and guarantees that any such liability is proportionate to the value of the claim.^50^ There are a multitude of cost protection rules that cap liability or confer immunity on certain classes of litigants to pay adverse cost orders if their claim is unsuccessful.^51^ There are disclosure rules directly designed to address information asymmetry between opposing parties, and which can help reduce the effects of resource asymmetry between the parties by requiring each party to hand over its evidence base to the other.^52^ There are (liberal) intervention rules, more commonly available in public law litigation, which allow representative organisations, non-governmental organisations, public bodies and even ordinary citizens to intervene in legal proceedings with the aim, or effect, of placing more or better arguments in support of an applicant’s position in litigation usually against the state or someone exercising powers conferred by the state.^53^ There are collective redress mechanisms, more commonly available in private litigation, designed to overcome any asymmetry of financial interest in the outcome of, and resources available to litigate, disputes affecting large numbers of people.^54^ There are specialist courts created with the aim of ensuring their procedures are specifically adapted to the needs of the litigants, with judges that have as much as or more expertise in the subject matter of the litigation than the lawyers appearing before them.^55^ We should also not overlook the overriding or overarching obligations on parties and lawyers to ensure that disputes are resolved proportionately.^56^ In some places, this proportionately requirement has been interpreted radically to include an obligation that parties are not over-represented.^57^

None of these procedural and funding solutions provide a complete answer to Wilmot-Smith’s thesis; if they did achieve perfect ‘equality of arms’ (to use ECtHR terminology), the market in legal services would look very different to what it does today. In practice, however, when these procedural innovations work well, and when they operate in combination, they can go a long way to levelling the playing field between parties who would otherwise be mismatched in the legal representation that they could obtain in a private market.

Wilmot-Smith glosses over other fundamental aspects of the legal process designed to ensure that those with the best lawyers do not win for that reason—most notably, the judge.^58^ At times reading Wilmot-Smith’s book, it feels as if the judge is powerless to prevent the rich skewing the benefits and burdens of legality in their favour. Wilmot-Smith would certainly deny that, so the argument boils down to how often it happens, and how much it could be ameliorated by better training of judges. There is a clear need to acknowledge that a judge should not remain passive, Sphinx like, while a well-resourced party runs rings around their opponent or litigates them into submission or bankruptcy. One doctrinal point that buttresses Wilmot-Smith’s concern is that while judges recognise that they need to be more interventionist when dealing with litigants in person, they continue to declare that they cannot even up the scales of justice by deploying their own legal expertise to assist an unrepresented or inadequately represented party. The fear is that this would involve judges entering the adversarial arena, rendering a trial unfair or creating an appearance of bias. In particular, judges are meant to refrain from entering the forensic, ie factual, arena, but they can clarify the parties’ legal positions.^59^ The difficulty with this approach is that the line between fact and law can be notoriously difficult to draw. When litigants are not in a position to identify the best legal arguments in support of their position, there is a risk that they might not adduce the appropriate evidence in support of those claims. These risks are particularly high in the case of unrepresented parties, but most lawyers will have witnessed cases where represented parties’ positions evolve considerably over the course of litigation, often in response to prompts and questions from judges. It is not obvious why a judge offering legal advice is permissible where both parties are represented by (roughly) equally experienced counsel, but constitutes a violation of their judicial impartiality where one party is unrepresented or clearly ‘outlawyered’. This brief dive into the detail shows that while there are plenty of difficulties thrown up by existing law to achieving equality of arms between litigants, there is also significant scope for improving procedural rules as a second-best solution for reducing the risk of unequal justice.

## 6. The Difficulty and Costs of Achieving Roughly Equal Legal Resources

Having briefly set out the equality gains that different procedural and funding rules might be able to achieve without the costs involved in Wilmot-Smith’s model, it is now worth considering ways in which Wilmot-Smith’s model might fall short of equal justice. A number of points could be made in this regard. Here again, I will run through them briefly, and in so doing sketch some of the potential benefits of tolerating a degree of inequality in access to legal resources.

First, it can be extremely difficult to eliminate from the courtroom the effects of any inequality that exist beyond it. One of the pressures on the legal aid budget that ultimately led to successive governments dramatically cutting it was the objective of not just providing legal representation to the poor, but also guaranteeing equal quality in legal representation, so that the poor were not at a disadvantage compared with their richer opponents.^60^ Wilmot-Smith’s proposal avoids the costs of levelling up, but there are also considerable practical challenges in levelling down by denying the rich the ability to buy legal services. Most obviously, the rich would still be able to buy access to unregulated legal services, including employing in-house counsel. Readily available access to in-house counsel provides considerable advantages both in litigation, even if the ‘lawyers’ have no rights of audience, and other advice contexts.^61^ Often, inequality in litigation is the product not of two mismatched barristers, but the level of additional legal support available to each party. For example, one senior barrister may be supported by a phalanx of external and in-house lawyers while the other senior barrister is instructed by a solitary lawyer with some paralegal support. Wilmot-Smith could not credibly force the phalanx outside the courtroom in a way that is consistent with the open justice principle, and even if it were possible, the inequality of arms would still usually show in the quality of the case presented in court. Wilmot-Smith recognises this risk, and that it could lead to a counsel of despair, but he also acknowledges that it shows why users of private legal services systems might be called upon to cross-subsidise the public system: the rich are still utilising the public system (in court proceedings) and degrading the quality of the public system (through the pull of entering the unregulated legal services market).^62^

Secondly, levelling down or up the legal talent available to parties in a dispute does not, of course, level the power available to those parties. This is particularly important in any form of investigation, dispute or proceeding involving the state and its regulatory agencies. One of the great benefits of Wilmot-Smith’s proposal is that it could make significant inroads into a crucial rule-of-law defect which is arguably increasing in size—the challenge of enforcement against corporations that have become so big that regulators cannot afford to take action against them and instead engage in forms of plea bargaining, but from a position of weakness rather than strength.^63^ On the other hand, public bodies possess a range of powers and privileges that are not available to even the richest citizens, including information-gathering powers and immunities from disclosure. One question I was left pondering after reading *Equal Justice* is whether the goal of providing ‘roughly equal legal resources’ also had implications for the asymmetry of procedural powers that is common in litigation involving the state and its representatives. It is plausible that Wilmot-Smith did not examine this subject, given that this is where the rules regarding the administration of justice begin to overlap with substantive public laws regulating the relationship between the state and citizen. Nonethless, clarification of his position on this issue would have been welcome.

Another related criticism of the resources Wilmot-Smith would dedicate to the equalisation of the legal services market is that it may not solve many of the causes of unequal justice. One cause is racism, a phenomenon that Wilmot-Smith references to demonstrate the influence of arbitrary factors on the justice system.^64^ There is undoubtedly intersectionality between race and class, but as a middle-class white person I am fully conscious of the fact that my odds of being stopped and searched by the police are vanishingly small compared to middle-class black people. While addressing wealth and race inequality are not mutually exclusive, greater judicial diversity and better implicit bias training, combined with other, less expensive and disruptive attempts to reduce the effects of wealth inequality, might produce more equality per pound (to use crude economic language) than what Wilmot-Smith’s proposals might be able to achieve.^65^

Finally, the diffuse nature of the legal services market makes achieving equality in access to legal services very difficult, and possibly undesirable. The diffuse nature of the legal services market is where the case for socialised healthcare and legal services start to diverge, even if they are helpful comparators in other respects. One of the strongest arguments in favour of socialised healthcare is that we are all human: we all have the same needs and same biological weaknesses, subject to relatively minor genetic variations. The body dictates the ranges of expertise required to run a successful healthcare system, and everyone may need to draw on that expertise at some point in their lives. The array of legal persons that a justice system must serve, and laws and legal claims it needs to administer, can be infinitely broader. Not all legal persons (real and artificial) need intellectual property lawyers, corporate lawyers or family lawyers, and so on. Although a primary focus of Wilmot-Smith’s book is equal justice in civil litigation and criminal proceedings, Wilmot-Smith does occasionally dive into the question of what equality demands of the legal services market generally. In particular, when discussing the question of taxation advice, Wilmot-Smith suggests that the best solution is to deny legal assistance to everyone on the grounds that taxation is principally concerned with welfare, not justice.^66^ This could lead to the problem mentioned above, where the rich can access unregulated legal services to assist them and the poor would get no assistance at all. More fundamentally, the non-litigitious legal services market is so diffuse it is not obvious what an equal legal advice service would look like.^67^

I am not suggesting that an account of an equal legal advice market is impossible, but it does require a definition of legal services, and at the margins this is not straightforward. In the *Three Rivers* litigation, which concerned the proper scope of legal advice privilege, Baroness Hale famously stated: ‘It is in the interests of the whole community that lawyers give their clients sound advice, accurate as to the law and sensible as to their conduct.’^68^ Legal advice privilege, therefore, was not confined to advice on the law, but extended to what should prudently and sensibly be done in a relevant legal context.^69^ A centralised system for dispensing this kind of ‘wise counsel’ would be complex and highly politicised. Moreover, the vast sums spent by the wealthy on such counsel (empirical studies suggest lawyers are virtually omnipresent at large corporations^70^), suggests that there is a high economic value in these services, and their allocation is likely to be efficient, given the sophisticated actors involved in the market. Rather than dedicate an enormous amount of effort to levelling the access to legal service available to corporations, the state could also choose to more effectively tax this market.

One argument in favour of this cross-subsidisation model is that the direct benefits of equalising the availability of lawyers can be marginal in a diffuse legal services market.^71^ Lawyers specialising in IPOs (initial public offerings) are not going to be much use to small businesses with no intention of going public. Suggesting IPO lawyers develop more general advice skills to assist ‘ordinary people’ with their everyday legal problems would be inefficient and reduce the level of specialist expertise.^72^ Of course, a centralised system need not mandate the training of only general-purpose lawyers; the NHS does not operate this way either. However, given that most of us will never need merger and acquisition and IPO lawyers, and that the people who do use them have enormous resources at their disposal and pay large amounts for them, there is a reasonable argument for using cross-subsidisation to better distribute the wealth generated by these services, as opposed to equalising access to them.^73^

The UK government has taken some tentative steps in the direction of cross-subsidisation of the justice system. In 2015, it introduced enhanced court fees, ie court fees levied above the costs to the public purse of resolving the dispute, for certain types of money claims. The fees were set at 5% of the total sums at issue. However, in a clearly regressive move, it capped such fees at £10,000 so that all disputes worth more than £200,000 were largely unaffected by the measure.^74^ One of the attractions of using enhanced court fees over a justice system funded entirely through general taxation is that they might be an effective way of taxing the many foreign companies and litigants who use the English justice system, but are not UK-domiciled taxpayers. Putting to one side the technical debate about the best way to maximise revenue from use of the justice system, and the precise shape of the Laffer curve for legal services,^75^ the key question is whether we wish to encourage a private legal services market and tax it more effectively to fund a more accessible system, albeit unequal in parts and with a higher risk of injustice in some cases, or eliminate it as part of a commitment to equal justice. Wilmot-Smith implicitly concedes that, all else being equal, his model would shrink the legal services market, which is why he couples his prohibition on negotiating private rates for legal services with a prohibition on contracting out of the public justice system altogether. His concern about a flight to arbitration by the wealthy is surely justified, but here, too, a second-best solution might be to find ways to better tax the private arbitration market, whether through taxing the use of the lawyers involved and/or through the court costs of enforcing arbitral awards.

Some of this equality-versus-efficiency argument can also be applied to arguments about healthcare. While a nationalised health system is achievable, whether it delivers greater health benefits overall than other systems is a matter of debate. This issue is far beyond this author’s expertise, but there is literature about whether mixed public and private models, such as the Australian system, or regulated, social-insurance-based models, common in Europe, might produce better health outcomes on some measures.^76^

## 7. Conclusion

Stepping back, it is important to acknowledge how modest my reservations about Wilmot-Smith’s model are. Even if they are all sound, they amount to a series of well-known but trite observations: no justice system is perfect; there is no single solution to all the faults of a justice system; all regulatory schemes lead to difficult cases at the margins; achieving equal distributions can lead to a reduction in the size of the goods being distributed; law is not the only urgent concern, and sometimes other concerns are more important.

The value of these points, combined with the procedural and funding rules that are, and could be, better designed to promote equal justice, is to highlight the key normative choice to be made: are we willing to pay the very substantial costs of reducing, perhaps modestly, the risk of unequal justice? Second-best solutions that retain a private legal service market will come with a higher risk of injustice (though we will never know how much higher). However, second-best solutions can produce substantial additional resources. The state could use these resources to deliver more justice benefits, by making the system more accessible to more people and for a wider range of legal problems, or it could use the taxation receipts to invest in other important public services.^77^ If we acknowledge the imperfect nature of even the fairest legal system—the dream of equal justice will only ever be a dream—the case for second-best solutions becomes more attractive.

To paraphrase Deng Xiaoping, if you cannot be sure the cat is catching and distributing the right mice to the right people, why not choose a cat that catches some mice for everyone, even if in unequal shares? There are two obvious flaws in this analogy which underline the strength of Wilmot-Smith’s book. First, our legal system does not currently deliver some justice to everyone; many are denied it completely. The second-best solutions I have sketched here are only slightly less of a pipe dream than Wilmot-Smith’s proposals for an equal justice system. Secondly, focusing on the wealth generated by a justice system, and the good works to which it can be put, clearly conflates justice with welfare benefits. This might be legitimate from a Marxist perspective, and particularly appealing to Chinese communist dictators whose regime is simultaneously responsible for lifting more people out of poverty than any other in history and presiding over a justice system that is a black hole. However, many readers and certainly most lawyers would regard a just legal system as a cornerstone of a just political order. Readers who accept that justice and welfare principles are not co-extensive will find this book compelling, and urgent, reading.

Perhaps Wilmot-Smith’s proposals will not be realised in practice, not because they lack merit, but rather because powerful forces would be aligned against them. Wilmot-Smith acknowledges this. But once upon a time, the task of creating a national health service that allocated medical care according to the value of the treatment to the patient, and not the ability of the patient to pay for it, in the face of vehement opposition from a conservative, well-organised profession, would have seemed impossible. I doubt we will see the day when right-wing politicians campaign against UK membership of co-operative regional or international institutions by promising to invest more in a nationalised legal service. If we do, however, Wilmot-Smith’s book will have played a small, important part in that transformation and I will be among the first to applaud him.

## Footnotes

1
*Equal Justice* 58.

2 ibid 96.

3 ibid 69. It can also distort the development of the common law, where certain types of disputes are more likely to be litigated in the higher courts, and the services of the best lawyers tend to be deployed in favour of certain (usually corporate) interests.

4 ibid 64: ‘The problem arises when powerful groups—those the law should aim to constrain—control the institutions (or access to the institutions) designed to hold them to account. When those groups are not able to mould the law to their whim, they can control its enforcement. They are able to renege on their legal obligations without risk of sanction, rendering any general rights that are granted a dead letter.’

5 ibid 106.

6
*Equal Justice* 74–5.

7 Whose pay would be determined in accordance with the best principles of distributive justice for allocating legal resources: ibid 173.

8 ibid 93.

9 The US Supreme Court has upheld such clauses as consistent with freedom-of-contract principles enshrined in arbitration statutes: see eg *Lamps Plus Inc v Varela* 587 US ____, 139 S. Ct. 1407, 1415 (2019); for criticism, see Judith Resnik, ‘Diffusing Disputes: The Public in the Private of Arbitration, the Private in Courts, and the Erasure of Rights’ (2015) 124 Yale LJ 2804. The extent to which ‘unfair’ arbitration clauses should be regulated by substantive contract law principles, such as the doctrine of unconscionability, or procedural principles that guarantee a right to a fair trial is a fascinating topic recently dealt with by the Canadian Supreme Court: *Uber Technologies Inc v Heller*, 2020 SCC 16.

10
*Equal Justice* 94.

11 See eg *Equal Justice* 125: ‘it is wrong to think of a just justice system as displacing individuals’ preferences … there is no way to implement a just justice system without centralised control … the decision to create [a legal system] is not an attempt to supplement individuals’ choices but to create a framework within which individuals are able to choose what they want’.

12 ibid ch 6.

13 In what reads to me as (interesting) detours, Wilmot-Smith also considers *where* justice should be administered as a way of opening up a discussion of online courts and tribunals and the importance of the open justice principle, and *how* judges should decide cases, ie the role of judges as law makers. These discussions, I think, warrant separate treatment in another book, and another review.

14 Myriam Gilles and Gary Friedman, ‘Examining the Case for Socialized Law’ (2020) 129 Yale LJ 2078, 2097.

15 See eg Alex Morales, ‘Boris Johnson Aims to Meet Brexit Bus Pledge with Boost for Health Care’ *Bloomberg* (30 July 2019) <www.bloomberg.com/news/articles/2019-07-30/johnson-aims-to-meet-brexit-bus-pledge-with-health-care-boost>.

16 Other reviewers have also noted that Wilmot-Smith does not examine the comparative case for his model against more limited reform options: Gilles and Friedman (n 14) 2110. This is not entirely accurate as Wilmot-Smith does look at the case for increasing civil legal aid for certain kinds of cases, but ultimately rejects it as being inadequate.

17 James Wilson, ‘Equal Justice: Fair Legal Systems in an Unfair World, by Frederick Wilmot-Smith’ [2020] Mind fzaa042<https://doi.org/10.1093/mind/fzaa042>.

18 John Rawls, *A Theory of Justice* (revised edn, Harvard UP 1999) 75.

19 ibid 74–5.

20
*Equal Justice* 22.

21 ibid 41, citing Michelle Alexander, *The New Jim Crow: Mass Incarceration in the Age of Colorblindness* (The New Press 2010) 96–7.

22
*Equal Justice* 107.

23 ibid ch 4.

24 ibid 106.

25 Ronald Dworkin, *A Matter of Principle* (Harvard UP 1985).

26 ibid 85: ‘Two people might be injured in precisely the same fashion by the same person. If one has a first-rate lawyer and the other a duffer, the first may get an adequate remedy and the second may get nothing. This would, the congruence condition shows, be a violation of the rule of law.’

27 Andrew Higgins, ‘The Rule of Law Case against Inconsistency and in Favour of Mandatory Civil Legal Process‘ (2019) 39 OJLS 725.

28 ibid.

29 For it introduces the risk of juries taking into account irrelevant information from other cases.

30
*Equal Justice* 77.

31 ibid 75: ‘In the adversarial context, the effectiveness of my lawyer depends on the effectiveness of yours.’

32 ibid 14.

33 ibid 3, quoting Thomas Nagel: ‘Justice plays a special role in political argument: to appeal to it is to claim priority over other values. Injustice is not just another cost; it is something that must be avoided, if not at all costs, then at any rate without counting the costs too carefully.’

34
*R (on the application of Unison) v Lord Chancellor* [2017] UKSC 51 [68].

35 In recent times, this was most famously articulated by Robert Nozick, *Anarchy, State and Utopia* (Basic Books 1974).

36 Wilmot-Smith observes that Nozick did not consider justice in the rectification of injustice, he simply said that injustices should be rectified: *Equal Justice* 217, fn 66, citing Nozick, ibid 151.

37 ibid 63.

38 ibid 43: ‘If the gains and losses of market transactions are to be justifiable to those who lose out, the legal structure must comply with equal justice.’

39 ibid.

40 See text to nn 33 and 34.

41 Jane Croft and Kate Beioley ‘Call to Give Companies “Breathing Space” on Coronavirus Litigation’ *Financial Times* (London, 26 April 2020).

42 ibid.

43
*Arkin v Marshall* [2020] EWCA Civ 620.

44 See eg G*older v UK* (1979–1980) 1 EHRR 524.

45 See eg *Kennedy v United Kingdom* (2011) 52 EHRR 4; *Home Office v Tariq* (2011) UKSC 35.

46 No doubt there could also be legal challenges to border closures, lockdowns, curfews etc.

47 A point also made by Wilson in his review (n 17) 8.

48
*Equal Justice* 74.

49 See the Damages Based Agreement Regulations 2013, which even the Ministry of Justice has acknowledged require reform to make them more widely available and economically viable: Post-Implementation Review of Part 2 of the Legal Aid, Sentencing and Punishment of Offenders Act 2012 (LASPO) Civil litigation funding and costs (February 2019) CP 38. Contingency fees are common in the United States and Canada, and there is a flourishing third-party litigation funding market in Australia.

50 Civil Procedure Rules (CPR) 45. Arguably the most comprehensive fixed fee regime is Germany’s Lawyer’s Remuneration Act (Rechtsanwaltsvergütungsgesetz, RVG) and Court Fee’s Act (Gerichtskostengesetz).

51 CPR 44.13–44.17 (Qualified One Way Cost Shifting) *R (Corner House Research) v. Secretary of State for Trade and Industry* [2005] EWCA Civ 192 (Protective Costs Orders); CPR 3.19–3.21 (Cost Capping Orders) The United Nations Economic Commission for Europe (UNECE) Convention on Access to Information, Public Participation in Decision-Making and Access to Justice in Environmental Matters.

52 CPR 31. It is in the public interest ‘that all relevant material should be available to courts when deciding cases. Courts should not have to reach decisions in ignorance of documents or other material which, if disclosed, might well affect the outcome’: *R v Derby Magistrates’ Court, ex p B* [1996] AC 487 [73].

53 In England, the court has the power to allow anyone to intervene in judicial review proceedings (CPR 54.17; see also CPR52.25) and is increasingly willing to do so. The same is also true of the UK Supreme Court (The Supreme Court Rules 2009, rules 15 and 26): see Mrs Justice Nathalie Lieven, ‘Interventions in Judicial Review Proceedings’ in A Higgins (ed), *The Civil Procedure Rules at 20* (OUP 2020) ch 7.

54 While opt-out class actions are available before the UK Competition Appeal Tribunal, the most mature class action regimes come from other common law countries, notably the United States (Rule 23 of the Federal Rules of Civil Procedure), Canada (eg Class Proceedings Act 1992 (Ontario)) and Australia (Federal Court of Australia Act 1976 (Cth), Part IVA).

55 This is a guiding principle of many specialist tribunals around the world, including England. To take just one example, the UK Competition Appeal Tribunal describes itself as ‘a specialist judicial body with cross-disciplinary expertise in law, economics, business and accountancy which hears and decides cases involving competition or economic regulatory issues’ <www.cattribunal.org.uk>.

56 CPR 1.1 (The Overriding Objective).

57 Civil Procedure Act 2010 (Victoria), ss 24, 29; Yara Australia Pty Ltd v Oswal [2013] VSCA 337.

58 Wilson (n 17) 8.

59 A recent case where the Employment Tribunal failed to do the latter for fear of doing the former is *Mervyn v BW Controls Ltd* [2020] EWCA Civ 393.

60 Adrian Zuckerman (ed), *Civil Justice in Crisis: Comparative Perspectives of Procedure* (OUP 1999) 36.

61 A point also made by Scheiber: ‘You can prevent the rich from hiring lawyers to represent them in an official legal capacity. But you can’t prevent them from hiring people with legal knowledge’: Noam Scheiber, ‘The Case for Socialized Law’ *New Republic* (3 February 2014) fn 21 <https://newrepublic.com/article/116424/socialized-law-radical-solution-inequality>.

62
*Equal Justice* 105.

63 One noteworthy example from Australia was a decision by the Competition and Consumer Regulator (the ACCC) to accept undertakings from international tobacco companies to no longer use ‘mild’ and ‘light’ descriptors in their marketing instead of taking enforcement action. The ACCC chairman defended the decision, stating: ‘A case like this is resource-intensive because of the nature of the evidence that is being brought … we would be fighting powerful companies with enormous resources and we have to consider that … On one analysis this could exhaust all our resources in one go.’ <www.tobaccoinaustralia.org.au/chapter-16-litigation/16-2-litigation-brought-by-australian-consumer-and-regulatory-groups-against-the-tobacco-industry>.

64 See n 21.

65 A similar point is made by Wilson in his review (n 17) 8.

66
*Equal Justice* 194: ‘few will be tempted by the thought that the government should supply [tax] lawyers; it just does not seem unfair to let individuals order their own tax affairs. This is best explained by the distinction between justice and welfare benefits. The tax system … is ultimately a matter of welfare …’

67 See eg Richard Abel, *Socializing the Legal Profession: Can Redistributing Lawyers’ Services Achieve Social Justice?* (1979) 1 Law & Policy 5, 15–16: ‘when we come to negotiation, planning, counseling, and drafting, the impossibility of equalizing access to legal representation hardly requires argument’.

68
*Three Rivers DC v Bank of England (No 6)* [2004] UKHL 48 [61].

69 ibid [62].

70 Vincent Alexander, ‘The Corporate Attorney–Client Privilege: A Study of the Participants’ (1989) 63 St John’s L Rev 191, 273.

71 Wilmot-Smith notes that even Marx was sympathetic to a user pays approach for legal services, given that they were then exclusively used by, and benefited, the ruling class: Karl Marx, *Critique of the Gotha Programme* (Robert C Tucker ed, 2nd edn, first published 1875, WW Norton & Company 1978) 539.

72 Wilmot-Smith agrees. He argues that any such pro bono scheme should require lawyers to pay some of their money, not their time, and that these resources should be reinvested in the justice system: *Equal Justice* 201.

73 For discussion on cross-subsidisation models, see Andrew Higgins, ‘Rebooting the Cab Rank Rule as a Limited Universal Service Obligation’ (2017) 20 Legal Ethics 201; Andrew Higgins, ‘The Costs of Civil Justice and Who Pays?’ (2017) 37 OJLS 687.

74 Ministry of Justice, *Enhanced Court Fees—The Government Response to Part 2 of the Consultation* (Cm 8971, 2015). The changes were introduced by The Civil Proceedings and Family Proceedings Fees (Amendment) Order 2015, SI 2015/576.

75 A point considered by the UKSC in *Unison*, though it was arguably misapplied in that case: see Andrew Higgins, ‘The Supreme Court Turns the Judicial Rhetoric on Access to Justice up to 11 As It Strikes Down Unaffordable and Disproportionate Employment Tribunal Fees, but Key Questions about the Funding of Civil Justice Remain: *R (on the application of Unison) v Lord Chancellor* [2017] UKSC 51’ (2018) 37 CJQ 1, 6–7.

76 See eg Irene Papanicolas and others, ‘Performance of UK National Health Service Compared With Other High Income Countries: Observational Study’ (2019) 367 British Medical Journal l6326 <http://dx.doi.org/10.1136/bmj.l6326>; The Commonwealth Fund, ‘Health Care System Performance Rankings’ (13 July 2017) <www.commonwealthfund.org/chart/2017/health-care-system-performance-rankings>. Wilmot-Smith’s thesis does not require complete equalisation of legal resources, but the fact that he asserts that equal justice requires a centralised process for allocating legal resources indicates that, for him, while such insurance-based or mixed public/private models might be more efficient, they would also produce more injustice.

77 Socialised systems, like all systems, must ration resources in some way. Wilmot-Smith sets out some guiding principles for determining the allocation of resources between cases, and people with legal problems, in a nationalised legal service in ch 10.

